# Draft Genome Sequence of Pelagicola sp. Strain LXJ1103, Isolated from Coastal Surface Seawater

**DOI:** 10.1128/MRA.00941-18

**Published:** 2018-10-11

**Authors:** Dexi Zhao, Jia Li, Shan Lin, Xuejiao Liang, Xiaopei Lin, Xueqiong Sun

**Affiliations:** a China National Offshore Oil Corporation Co., Ltd., Tianjin Branch, Tianjin, China; b State Key Laboratory of Marine Environmental Science, Institute of Marine Microbes and Ecospheres, Xiamen University, Xiamen, China; University of Arizona

## Abstract

Pelagicola sp. strain LXJ1103, a representative of a new species in the family Rhodobacteraceae, was isolated from the coastal surface waters in Xiamen, China. Here, we announce the draft genome sequence and initial findings from a preliminary analysis of strain LXJ1103.

## Abstract (precis)

Pelagicola sp. strain LXJ1103, a representative of a new species in the family Rhodobacteraceae, was isolated from the coastal surface waters in Xiamen, China.

## ANNOUNCEMENT

Pelagicola sp. strain LXJ1103 (CGMCC 1.15911), displaying a red color, was isolated from coastal surface waters (24.60°N, 118.16°E; depth, 5 m) near Xiamen, China. We collected a seawater sample, which was suspended in 20.0% (vol/vol) glycerol and stored at −80°C. Isolation was performed using the standard dilution plating technique. A 100-μl suspension was spread on marine agar medium (catalog number 2216E; Difco, USA) and cultured at 25°C for 1 week, after which the red colony was picked and purified. Comparative 16S rRNA gene sequence analysis indicated that strain LXJ1103 is phylogenetically related most closely to members of the genus Pelagicola within the family Rhodobacteraceae (Roseobacter clade). As shown previously, members of the Rhodobacteraceae are the dominant bacteria found in the biofilm of eastern Mediterranean coastal seawater, regardless of season (1). Members of the genus Pelagicola are Gram negative, strictly aerobic, and nonmotile and have the form of club-shaped rods (2). Here, we report the draft genome sequence of strain LXJ1103.

In this project, genomic DNA was extracted and purified from cells cultivated in marine agar medium for 2 days at 25°C using a Tguide bacterial genomic DNA kit (catalog number OSR-M502; Tiangen Biotech, China) following the manufacturer’s instructions. The genome-wide shotgun strategy was used to construct libraries of different inserts. This strain was sequenced on the MiSeq platform (Illumina, Inc., USA) following the preparation of a library of paired-end sequences. The reads were assembled using the A5-miseq version 20150522 pipeline (3). The total number of Illumina reads was 2,423,720, and the coverage was 176×. The draft genome of strain LXJ1103 comprised one linear chromosome of 3,040,865 bp, distributed in two scaffolds, and had a G+C content of 60.98%. Genes were annotated using the Evolutionary Genealogy of Genes: Nonsupervised Orthologous Groups (eggNOG) (4), Kyoto Encyclopedia of Genes and Genomes (KEGG) (5), and Gene Ontology (6) databases. Putative functions from functional clusters of orthologous groups (COG) were assigned to 91.91% of the candidate genes, and RpsBLAST version 2.2.30+ was used to predict the protein sequence. A total of 3,048 genes were predicted, and 2,961 of them were protein-coding genes. We predicted the tRNAs and rRNAs using tRNAscan-SE version 1.31 (7) and RNAmmer version 1.2 (8), respectively. Totals of 38 tRNAs, 3 rRNA operons, 3 other RNAs, and 43 pseudogenes were found in the genome.

Strain LXJ1103 contained an abundance of ATP-binding cassette system (ABC transporter) genes in the genome, through which the hydrolysis of bacteria leads to the translocation of various genetic substrates across membranes, either for uptake or for the export of the substrate (9). Genes putatively encoding the biosynthesis, export, and transport of capsular polysaccharides were also identified in the genome. Strain LXJ1103 possessed genes responsible for a carbon monoxide dehydrogenase. Genes for sulfur metabolism, including sulfate transporter, sulfate permease, sulfate adenylyltransferase, and phosphoadenosine phosphosulfate reductase, were also present in the strain LXJ1103 genome. Analysis with PHAST (PHAge Search Tool) software identified one intact prophage region in the genome (10).

### Data availability.

The shotgun sequence of Pelagicola sp. strain LXJ1103 has been deposited at GenBank under the accession number QFAR00000000. The version described in this paper is the ﬁrst version, QFAR01000000. The SRA accession number is SRP159213.
